# Co-occurring nitrifying symbiont lineages are vertically inherited and widespread in marine sponges

**DOI:** 10.1093/ismejo/wrae069

**Published:** 2024-04-27

**Authors:** Bettina Glasl, Heidi M Luter, Katarina Damjanovic, Katharina Kitzinger, Anna J Mueller, Leonie Mahler, Joan Pamela Engelberts, Laura Rix, Jay T Osvatic, Bela Hausmann, Joana Séneca, Holger Daims, Petra Pjevac, Michael Wagner

**Affiliations:** Division of Microbial Ecology, Centre for Microbiology and Environmental Systems Science, University of Vienna, 1030 Vienna, Austria; Australian Institute of Marine Science, 4810 Townsville, Australia; Australian Institute of Marine Science, 4810 Townsville, Australia; Division of Microbial Ecology, Centre for Microbiology and Environmental Systems Science, University of Vienna, 1030 Vienna, Austria; Division of Microbial Ecology, Centre for Microbiology and Environmental Systems Science, University of Vienna, 1030 Vienna, Austria; Doctoral School in Microbiology and Environmental Science, University of Vienna, 1030 Vienna, Austria; Division of Microbial Ecology, Centre for Microbiology and Environmental Systems Science, University of Vienna, 1030 Vienna, Austria; Australian Centre for Ecogenomics, School of Chemistry and Molecular Biosciences, The University of Queensland, 4072 St. Lucia, Australia; Centre for Microbiome Research, School of Biomedical Sciences, Queensland University of Technology, Translational Research Institute, 4102 Woolloongabba, Australia; Australian Centre for Ecogenomics, School of Chemistry and Molecular Biosciences, The University of Queensland, 4072 St. Lucia, Australia; Joint Microbiome Facility of the Medical University of Vienna and the University of Vienna, 1030 Vienna, Austria; Division of Clinical Microbiology, Department of Laboratory Medicine, Medical University of Vienna, 1090 Vienna, Austria; Joint Microbiome Facility of the Medical University of Vienna and the University of Vienna, 1030 Vienna, Austria; Division of Clinical Microbiology, Department of Laboratory Medicine, Medical University of Vienna, 1090 Vienna, Austria; Division of Microbial Ecology, Centre for Microbiology and Environmental Systems Science, University of Vienna, 1030 Vienna, Austria; Joint Microbiome Facility of the Medical University of Vienna and the University of Vienna, 1030 Vienna, Austria; Division of Microbial Ecology, Centre for Microbiology and Environmental Systems Science, University of Vienna, 1030 Vienna, Austria; Division of Microbial Ecology, Centre for Microbiology and Environmental Systems Science, University of Vienna, 1030 Vienna, Austria; Joint Microbiome Facility of the Medical University of Vienna and the University of Vienna, 1030 Vienna, Austria; Division of Microbial Ecology, Centre for Microbiology and Environmental Systems Science, University of Vienna, 1030 Vienna, Austria; Joint Microbiome Facility of the Medical University of Vienna and the University of Vienna, 1030 Vienna, Austria; Department of Chemistry and Bioscience, Aalborg University, 9220 Aalborg, Denmark

**Keywords:** sponge microbiome, ammonia-oxidizing archaea, nitrite-oxidizing bacteria, symbiont transmission, *Candidatus* Nitrosokoinonia gen. nov, *Candidatus* Nitrosymbion gen. nov

## Abstract

Ammonia-oxidizing archaea and nitrite-oxidizing bacteria are common members of marine sponge microbiomes. They derive energy for carbon fixation and growth from nitrification—the aerobic oxidation of ammonia to nitrite and further to nitrate—and are proposed to play essential roles in the carbon and nitrogen cycling of sponge holobionts. In this study, we characterize two novel nitrifying symbiont lineages, *Candidatus* Nitrosokoinonia and *Candidatus* Nitrosymbion in the marine sponge *Coscinoderma matthewsi* using a combination of molecular tools, *in situ* visualization, and physiological rate measurements. Both represent a new genus in the ammonia-oxidizing archaeal class *Nitrososphaeria* and the nitrite-oxidizing bacterial order *Nitrospirales*, respectively. Furthermore, we show that larvae of this viviparous sponge are densely colonized by representatives of *Ca*. Nitrosokoinonia and *Ca*. Nitrosymbion indicating vertical transmission. In adults, the representatives of both symbiont genera are located extracellularly in the mesohyl. Comparative metagenome analyses and physiological data suggest that ammonia-oxidizing archaeal symbionts of the genus *Ca*. Nitrosokoinonia strongly rely on endogenously produced nitrogenous compounds (i.e. ammonium, urea, nitriles/cyanides, and creatinine) rather than on exogenous ammonium sources taken up by the sponge. Additionally, the nitrite-oxidizing bacterial symbionts of the genus *Ca*. Nitrosymbion may reciprocally support the ammonia-oxidizers with ammonia via the utilization of sponge-derived urea and cyanate. Comparative analyses of published environmental 16S rRNA gene amplicon data revealed that *Ca*. Nitrosokoinonia and *Ca*. Nitrosymbion are widely distributed and predominantly associated with marine sponges and corals, suggesting a broad relevance of our findings.

## Graphical Abstract

**Graphical Abstract:**
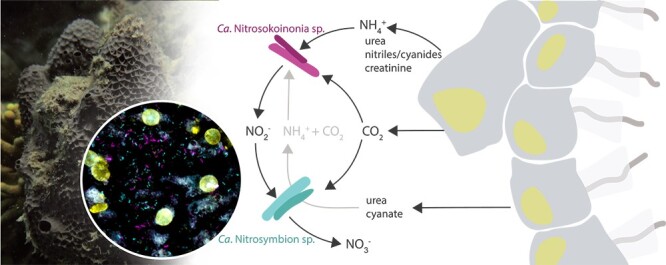

## Introduction

Marine sponges (phylum Porifera) are amongst the oldest metazoans [1]. They evolved around 650 million years ago [2]. The sessile filter-feeding lifestyle and the ability to take up and recycle dissolved organic matter (DOM) via the so-called sponge loop make sponges important members of almost all marine benthic ecosystems, particularly in oligotrophic regions such as coral reefs [3–5]. Their evolutionary and ecological success can partly be attributed to the diverse metabolic interactions with the abundant microbes they harbour in their tissue [6–8]. Despite their ability to filter large amounts of seawater resulting in constant exposure to environmental microbes [9, 10], sponges discriminate between symbionts and food extremely well [11]. Consequently, each sponge species is equipped with a distinct and relatively stable microbiome [12], which is mostly passed vertically from one generation to the next, but horizontal acquisition has also been reported [11, 13]. In an adult sponge, the microbiome can contribute up to 35% of the sponge volume [14, 15] and facilitates key processes such as the removal of nitrogenous waste products, the production of vitamins and amino acids, and the uptake of DOM [6, 16–19]. Overall, the symbiotic interactions of marine sponges with their microbiomes can be seen as one of the evolutionarily earliest examples of an animal holobiont, providing key insights into the origin of animal–microbe symbioses [20].

Nitrification, the oxidation of ammonia (NH_3_) to nitrite (NO_2_^−^) and further to nitrate (NO_3_^−^), is a key process in many marine sponge holobionts and is exclusively mediated by their microbial symbionts (e.g. [6, 21–28]). Ammonium (NH_4_^+^) is a metabolic waste product of the sponge and has a higher toxicity to aquatic animals than nitrite and nitrate [29]. The symbiont-driven transformation of ammonium is thus essential for the host. Additionally, the growth of nitrifiers might also represent an efficient recycling strategy to secure microbial biomass as a food source for the sponge [17]. Ammonia-oxidizing archaea (AOA), class *Nitrososphaeria*, are key players in the nitrification process in marine sponges [23, 30–34]. Ammonia-oxidizing bacteria (AOB) can also be found in sponges, yet they play only a minor role compared to AOA [23, 33, 35]. The second step of nitrification in sponges is mediated by nitrite-oxidizing bacteria (NOB), predominantly of the order *Nitrospirales* [6, 16, 36]. Both ammonia- and nitrite-oxidizers are chemolithoautotrophs and use the energy gained from the oxidation of ammonia or nitrite, respectively, to fix dissolved inorganic carbon (DIC) [37, 38]. Despite the frequent detection of putative nitrifying sponge symbionts using amplicon and metagenome sequencing [6, 16, 22, 31, 36, 39], studies investigating their contribution to the health and nutrition of the host and their activity and localization within the sponge tissue over different life stages remain scarce [17, 21, 23, 25].

In this study, we characterized the environmental distribution, metabolic potential, metabolic function, and transmission mechanisms of nitrifying symbionts associated with the tropical marine sponge *Coscinoderma matthewsi,* using a combination of physiological rate measurements, molecular tools, and epifluorescence microscopy. The ammonia- and nitrite-oxidizing symbionts of *C. matthewsi* belong to yet-uncharacterized novel genera of the archaeal family *Nitrosopumilaceae* and the bacterial order *Nitrospirales*, respectively. Members of both genera seem to be predominantly associated with marine sponges and corals, frequently co-occur within the same host individual, and appear to be vertically transmitted. This study provides a comprehensive assessment of the symbiotic association and metabolic function of these novel AOA and NOB lineages ubiquitously associated with marine sponges.

## Materials and methods

### Sponge collection

Three sponge individuals of the species *C. matthewsi* were collected at 8 m depth from the reef surrounding Falcon Island (18° 45' 59.3" S, 146° 32' 7.8" E, Great Barrier Reef, Australia) in October 2021. Sponge species identification was confirmed by amplification and sequencing of two host phylogenetic marker genes (see Supplementary Methods and Supplementary Figs S1 and S2). To ensure minimal impact on the natural sponge population, only parts of each sponge individual were removed, leaving the remaining sponge (at least one third) on the reef. Sponges were collected under permit G21/38062.1 issued by the Great Barrier Reef Marine Park Authority. Removed sponge fragments were immediately relocated to a flow-through aquaria system at the National SeaSimulator at the Australian Institute of Marine Science (Townsville, Australia). After a one-month acclimation period, each sponge individual (*n* = 3) was fragmented into three equally sized explants (~5 cm in diameter) using sterile scalpel blades. Sponge explants (*n* = 9) were allowed to heal in an outdoor flow-through aquaria system (2500 L) under natural lighting and ambient seawater temperatures (~27°C) for two weeks before further experiments.

### Experimental setup

Nitrification activity of the *C. matthewsi* sponge holobiont was assessed during a six-hour incubation experiment. Sponge explants (each containing at least one visible osculum) were placed in 1.5 L acid-washed glass jars prefilled with 1 L of 0.1 μm filter-sterilized (Sawyer, hollow fibre membrane filter) seawater. One explant from each sponge individual (*n* = 3) was subjected to one of the three different treatment conditions: (i) ambient NH_4_^+^, (ii) 5 μM NH_4_^+^, and (iii) 25 μM NH_4_^+^; resulting in three explants incubated at each condition. In the “ambient NH_4_^+^” treatment, no additional ammonium source was added, and the sponge explants were exposed to ambient ammonium concentrations of in-shore reef sites (~0.10 μM). In the 5 and 25 μM NH_4_^+^ treatments, the ammonium concentration was adjusted accordingly by addition of ammonium chloride (NH_4_Cl) from a sterile 25 mM stock solution before sponge explants were placed into the incubation jars. The elevated ammonium treatments reflect the approximate toxicity threshold for aquaculture species (100 μg L^−1^ ammonia) and the toxicant trigger value in marine water (900 μM L^−1^ ammonia) [40]. In addition, abiotic control incubations (1 L of 0.1 μm filter-sterilized seawater (FSW) without a sponge explant) were carried out in triplicates for each of the three treatment conditions. All incubation jars (*n*  = 18) were randomly distributed amongst three chest-type orbital shaker incubators (Thermoline Scientific). Each incubator was set to 60 rpm and 27.5°C, matching the *in situ* seawater temperature. All jars were kept in the dark throughout the experiment, as *C. matthewsi* is a nonphototrophic sponge. A HQ30D portable multimeter and an Intellical LDO101 oxygen probe (Hach, CO, USA) were used to measure oxygen concentrations at 0 and 6 h as proxy for sponge holobiont respiration. Oxygen levels at the end of the experiment (6 h) remained at 79% (±5% SD) and 99% (±1% SD) saturation in the sponge and abiotic control incubations, respectively.

Samples for determining nitrification activity were collected at four time points during the incubation (0, 1, 3, and 6 h). At each time point, 30 ml of seawater was sampled using a 50 ml syringe and subsequently filtered through a 0.45 μm filter (Sartorius Minisart, cellulose acetate membrane). Discarding the initial 10 ml of filtrate, the remaining 20 ml were divided into duplicates and stored at −20°C until further processing. Nitrite, nitrate, and ammonium concentrations in the samples were submitted to the AIMS analytical centre (Townsville, Australia) and analyzed according to their standard operating procedures [41] against OSIL standards and in-house reference samples using a Seal AA4 segmented flow analyzer (Seal Analytical).

After the last sampling time point (6 h), the wet weight (ww) of each sponge explant was measured, and the sponge tissue was then rinsed with 0.1 μm FSW to remove loosely attached microbes. Subsequently, the tissue was subsampled for molecular analyses and microscopy. In addition, the explants of two out of three sponge individuals released larvae during the incubation experiments (~2 h into the experiment). Newly released larvae from the “ambient NH_4_^+^” and “5 μM NH_4_^+^” incubation jars were collected (~2 h after the release) using 1 ml plastic pipettes, pooled per individual, and processed for molecular analysis and microscopy.

### 16S rRNA gene amplicon sequencing and analysis

Subsampled sponge tissue incubated under “ambient NH_4_^+^” conditions (*n*  = 3) was snap-frozen in liquid nitrogen and pooled larvae samples from two sponge individuals (*n* = 4, each sample containing four to six larvae) were preserved in molecular-grade absolute ethanol. In addition, 1 L of 0.1 μm freshly FSW (*n*  = 2) was filtered onto a 0.2 μm Sterivex (Merck Pty Ltd) filter and snap-frozen in liquid nitrogen. All samples were stored at −80°C until the DNA was extracted using the DNeasy PowerSoil Pro Kit (Qiagen). A blank DNA extraction was included to account for possible contaminations. The V4 region of the 16S rRNA gene was amplified and sequenced on a MiSeq system (Illumina) at the Joint Microbiome Facility (JMF) of the Medical University of Vienna and the University of Vienna, using the primer pair 515F 5′-GTG YCA GCM GCC GCG GTA A-3′ [42] and 806R 5′-GGA CTA CNV GGG TWT CTA AT-3′ [43], as previously described [44] (see Supplementary Methods for further details). Amplicon sequence variants (ASVs) were inferred from demultiplexed amplicon data in pooled mode with default settings with the DADA2 R package v1.20.0 (R 4.1.1) [45, 46] and classified with the SINA classifier v1.6.1 [47] against the SILVA reference database (SILVA release 138). ASVs classified as chloroplast and mitochondria were removed from the ASV table. Singletons (reads that occur only once) were removed from the ASV table. and reads were rarefied to an equal sequencing depth of 4424 sequencing reads for the subsequent data analysis.

Alpha diversity measures (i.e. richness, evenness, and Shannon index) were calculated using the phyloseq package [48]. Data analysis and figure generation were performed in R [49] using the packages dplyr [50] and ggplot2 [51], respectively.

### Metagenome sequencing, *de novo* assembly, and binning

DNA of one pooled larvae sample (larvae #2a) was selected based on the relative abundance of nitrifying symbionts revealed by 16S rRNA gene amplicon sequencing and was submitted to the JMF for metagenome sequencing in paired-end mode (2× 100 bp) on a NovaSeq SP system (Illumina; see Supplementary Methods for further details). Metagenomic data were assembled using metaSPAdes v3.15.5 [52] with kmers set to 21, 31, 41, 51, 61, 71, 81, 91, 101, 111, 121. Contigs with <1000 bp were removed from the assembly.

The assembly was binned into metagenome assembled genomes (MAGs) using MetaBAT2 v2.15, run with a minimum contig length of 1500 bp and default workflow was used in concoct [53]. Coverage information was calculated using BBMap v39.01 with 98% identity, and the resulting bam file was sorted using samtools v1.17. MAGs were also binned using MetaBAT2 with no coverage information. Both resulting MAG sets were dereplicated using dRep v3.4.2 [54]. Completeness and contamination of the MAGs were calculated using dRep’s checkM output [55]. Coverage for high-quality MAGs was calculated using BBMap at 95% mapping identity.

### Phylogenetic placement of metagenome-assembled genomes

The taxonomy of recovered high-quality (>90% complete and <5% contamination) MAGs [56] was assigned using GTDB-tk v2.3.2 [57]. MAGs belonging to the archaeal family *Nitrosopumilaceae* (*n* = 1) and the bacterial order *Nitrospirales* (*n* = 1) were screened for 16S rRNA genes using Barrnap v0.9, and the recovered genes were aligned against the representative ASV sequences of the 16S rRNA gene amplicon dataset with the nucleotide blast tool [58]. In case of the archaeal MAG, no 16S rRNA gene was detected; therefore, the 16S rRNA gene amplicon reads of the dominant archaeal ASVs were placed into a full-length 16S rRNA gene tree of publicly available AOA genomes with pplacer v1.1alpha19 [59]. Phylogenomic trees of MAGs generated in this study and of publicly available genomes of closely related organisms (>90% complete and <5% contamination; Supplementary Table S1) were constructed using concatenated amino acid sequences of marker proteins extracted using checkM v1.1.3 [55]. Phylogenetic trees were constructed in IQ-TREE v2.1.2 [60], see Supplementary Methods for details, and visualized in iTOL v5 [61].

### Global distribution and co-occurrence of novel nitrifying symbiont lineages

The global distribution and prevalence of the novel nitrifying archaeal and bacterial lineages were investigated by screening publicly available 16S rRNA gene amplicon sequencing datasets with the integrated microbial next-generation sequencing (IMNGS) platform [62]. Full-length 16S rRNA gene sequences of the recovered MAGs (or their close relatives) were submitted as a “parallel similarity” query against all available sequences in IMNGS (as of October 2023) with a similarity threshold of 97%, a minimum length of 200 nucleotides, and a minimal relative abundance of 0.01%. The similarity threshold was chosen based on the 16S rRNA gene sequence similarities amongst members of the novel nitrifying archaeal and bacterial lineages (Supplementary Table S1). IMNGS habitat classifications were manually corrected by cross-checking to original NCBI Sequence Read Archive entries. The following three categories were then assigned to the samples: “sponge,” “coral,” and “other” (Supplementary Table S2). The data were analyzed and visualized using the R packages dplyr [50] and ggplot2 [51].

### Metagenome assembled genome annotation

Nitrifier MAGs from this study (*n* = 2) and a dereplicated set of related, publicly available MAGs and isolate genomes (*n* = 82; Supplementary Table S1) were functionally annotated using the eggnog mapper v2.1.10 [63] and the MicroScope platform v3.16.0 [64]. Genome features (i.e. completeness, contamination, genome size, GC content, coding densities, and the number of predicted genes) were assessed using checkM v1.1.3 [55]. The annotations were manually screened for genes of key metabolic features of AOA and NOB (i.e. CO_2_ fixation, ammonia and nitrite oxidation, electron transport, and alternative substrate utilization). Unique and shared orthologs (based on “eggNOG OGs”) amongst MAGs/genomes were identified and visualized using the R packages dplyr [50] and Venn diagram [65].

### Absolute quantification of nitrifying symbionts

Absolute abundances of the dominant AOA and NOB in the sponge tissue and larvae samples were quantified with droplet digital PCR (ddPCR) and symbiont specific primer sets using the Bio-Rad QX200 system (Bio-Rad Laboratory). Detailed description on the primer design, ddPCR optimization, and ddPCR amplification is provided in the Supplementary Methods. The resulting concentrations (copies μl^−1^) were corrected for the DNA template dilution factor and normalized to the wet weight (copies g^−1^ ww) of the sponge tissue and larvae used for DNA extraction (Supplementary Methods).

### Visualization of nitrifying symbionts using fluorescence *in situ* hybridization

Freshly collected sponge tissue and larvae were fixed in 4% paraformaldehyde (ProSciTech) overnight at 4°C, rinsed three times with ice-cold 1× phosphate-buffered saline (PBS), and subsequently stored in PBS:ethanol (1:1) at −20°C. Thin sections of sponge tissue were prepared at the Histology Facility of the Vienna Biocenter (Vienna, Austria). In brief, fixed samples were washed for 1 h in 1× PBS and stored overnight at 4°C in 30% sucrose to preserve tissue morphology. Samples were subsequently transferred to 50% sucrose:Tissue-Tek O.C.T compound embedding medium (Sakura) for embedding, and stored overnight at 4°C. The embedded samples were transferred to isopentane and frozen in liquid nitrogen. Embedded tissue blocks were kept either on dry ice or at −80°C, until 5 μm-cryosections were prepared.

The 16S rRNA-targeted oligonucleotide probe Arch915 [66], tetra-labelled [67] with fluorochrome Atto565 (Biomers), was used for visualization of AOA in tissue sections of *C. matthewsi*. *Bacteria* belonging to the genus *Nitrospira* were visualized using the 16S rRNA-targeted probe Ntspa662 [68] double-labelled [69] with Cy5 (Biomers). Hybridizations (4 h at 46°C) were performed with an equimolar mixture of both probes (0.5 μM each) and 30% formamide (v/v) in the hybridization buffer. The stringency of the washing buffer was adjusted accordingly [70]. Additional tissue sections were hybridized with probe non-EUB338-I [71] tetra-labelled with Atto565 or double-labelled with Cy5 and served as negative controls (Supplementary Fig. S3). Sequences of fluorescence *in situ* hybridization (FISH) probes are provided in Supplementary Methods. All hybridized tissue sections were 4′,6-diamidino-2-phenylindole (DAPI)-stained and subsequently visualized at 1000× magnification on a THUNDER Leica Imaging System.

## Results

### Microbiome composition and vertical symbiont transmission in *C. matthewsi*

**Figure 1:**
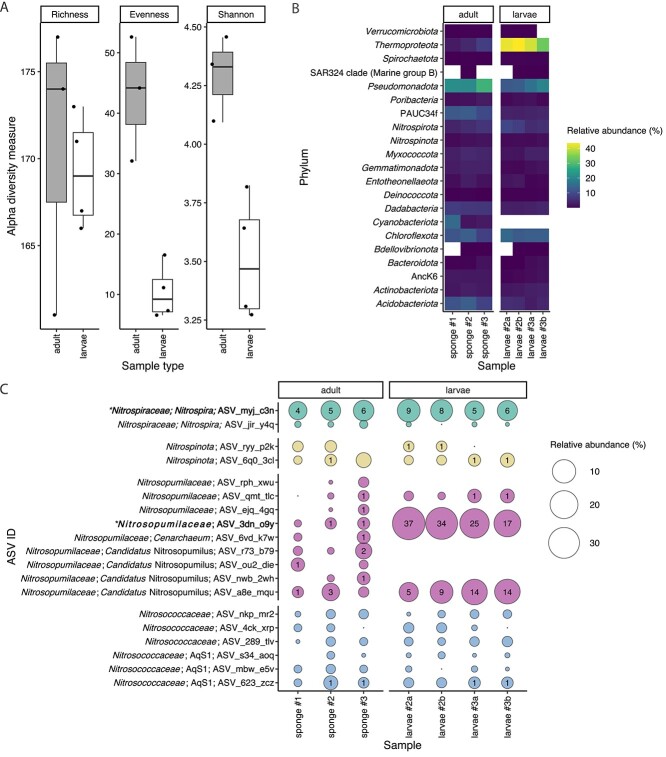
Alpha diversity and taxonomic composition of the *C. matthewsi* microbiome. (A) Microbiome richness, evenness, and Shannon diversity associated with adult and larvae samples. (B) Bacterial and archaeal phyla (based on GTDB taxonomy) detected by 16S rRNA gene amplicon sequencing of adult sponge tissue (individual #1–#3) and pooled larvae samples (recovered from sponge individual #2 and #3). (C) Relative abundance of individual ASVs of putative nitrifying symbionts associated with adult sponge tissue and larvae samples. The asterisk (^*^) highlights ASVs for which representative high-quality MAGs were obtained.

On average, 171 (±9 SD) unique ASVs were detected by 16S rRNA gene amplicon sequencing in the tissue of the sponge *C. matthewsi*. An equally high richness of unique ASVs was present in the freshly released larvae (169 ± 3 SD), and most ASVs were detected in the adult sponge as well as corresponding larvae (Supplementary Fig. S4). The skewed evenness of the larval microbiome, however, led to a reduced alpha diversity (based on the Shannon index) in the larvae (3.51 ± 0.27 SD) compared to the adult tissue (4.29 ± 0.18 SD) samples (Fig. 1A). Overall, 21 (±1 SD) distinct microbial phyla were detected in the adult sponge tissue and 20 (±1 SD) microbial phyla in the larvae (Fig. 1B) samples. *Cyanobacteriota* was the only microbial phylum that was consistently absent in the larvae when compared to the adult sponge microbiome (Fig. 1B and Supplementary Fig. S4). Furthermore, the larval microbiome was dominated (39.7% ± 4.7% SD) by ASVs belonging to putative ammonia-oxidizing archaeal symbionts of the phylum *Thermoproteota* (syn. *Crenarchaeota*), family *Nitrosopumilaceae* (Fig. 1B and C). In contrast, the same ASVs only made up on average 5.0% (±2.6% SD) of the microbiome associated with the tissue of adult sponges.

**Figure 2:**
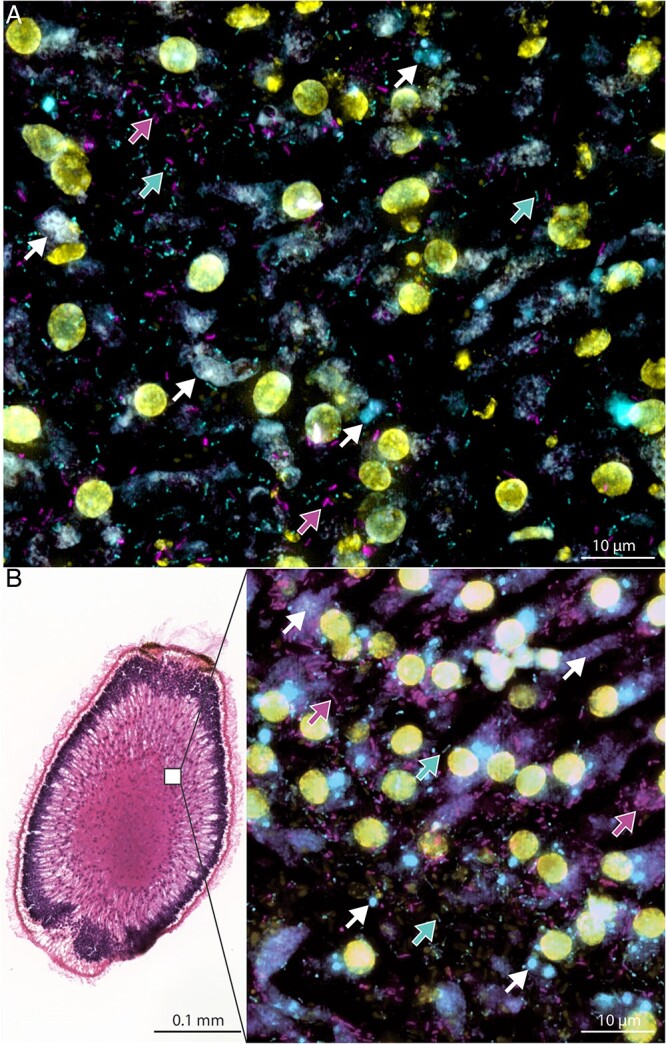
Visualization of nitrifying symbionts in the sponge *C. matthewsi* using FISH. Symbionts were visualized in 5 μm cryosections of (A) adult sponge tissue and (B) freshly released larvae (longitudinal section of entire larva). Archaeal symbionts (based on 16S rRNA gene amplicon sequencing all belonging to the ammonia-oxidizing family of *Nitrosopumilaceae*) were hybridized using a tetra-labelled Arch915 probe (Atto565, shown in magenta, see magenta arrows). Putative nitrite-oxidizing symbionts of the order *Nitrospirales* were hybridized using the double-labelled Ntspa662 probe (Cy5, shown in cyan, see cyan arrows). Eukaryotic and prokaryotic DNA (if not additionally stained with an FISH probe) was stained using DAPI (shown in yellow). Purple/blue and white structures represent the autofluorescence of sponge tissue structures (see white arrows).

Cumulatively, putative nitrifying ASVs (function inferred based on their taxonomy; “*Nitroso*” = ammonia oxidizers, “*Nitro*” = nitrite oxidizers) made up on average 13.1% (±4.7% SD) and 49.1 (±5.7% SD) of the microbiome associated with adult sponges and larvae, respectively (Fig. 1C). In general, the putative ammonia-oxidizing community (“*Nitroso”*) was dominated by two ASVs (ASV_3dn_o9y and ASV_a8e_mqu) belonging to the archaeal family *Nitrosopumilaceae*; both were present in all sequenced adult and larvae samples and were found in high relative abundances in all larvae samples. Absolute quantification with symbiont-specific ddPCR primers targeting the archaeal symbiont with the highest relative abundance (ASV_3dn_o9y) revealed an average absolute abundance of 1.02 (±0.83 SD) × 10^6^  *amoA* gene copies g^−1^ ww sponge tissue and 1.97 (±1.54 SD) × 10^9^  *amoA* gene copies per g^−1^ ww larva. The nitrite-oxidizing community (“*Nitro”*) was dominated by one ASV (ASV_myj_c3n) of the bacterial order *Nitrospirales*, which also was present in all adult and larvae samples. Absolute quantification using symbiont-specific ddPCR primers targeting the 16S rRNA gene of the dominant *Nitrospira* symbiont revealed an average absolute abundance of 4.90 (±4.00 SD) × 10^7^ 16S rRNA gene copies g^−1^ ww sponge tissue and 1.43 (±0.94 SD) × 10^9^ 16S rRNA gene copies per g^−1^ ww larva. Furthermore, the AOA as well as nitrite-oxidizing symbionts of the order *Nitrospirales* were visualized using FISH (Fig. 2). Both symbiont clades were present in high abundance in the extracellular medium (mesohyl) of adult sponges, and in the central mesohyl of freshly released larvae.

### Nitrification rates of the sponge holobiont

**Figure 3:**
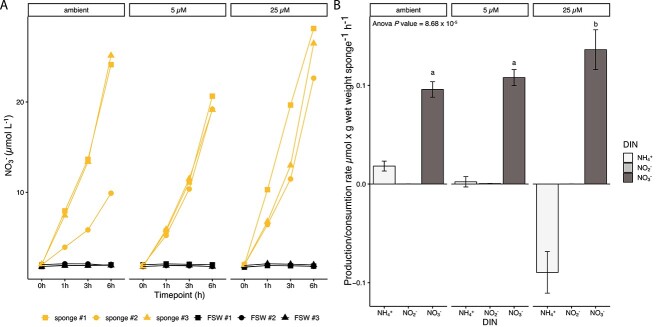
Nitrification activity of the *C. matthewsi* holobiont. (A) Production of nitrate (NO_3_^−^) per sponge individual and in abiotic control incubations in 0.1 μm FSW during the 6 h incubation experiment under ambient and amended ammonium (NH_4_^+^) concentrations (5 and 25 μM, respectively). (B) DIN production (>0) and consumption (<0) rates per gram wet weight (ww) sponge per hour. Rates and standard errors (represented as error bars) were calculated with a linear regression model across all time points. Ammonium (NH_4_^+^), nitrite (NO_2_^−^), and nitrate (NO_3_^−^) production/consumption rates are displayed for each of the three treatment conditions (i.e. ambient, 5, and 25 μM NH_4_^+^).

Nitrification rates of the *C. matthewsi* holobiont were experimentally determined to verify the ammonia- and nitrite-oxidizing capacity of the associated microbiome. Nitrite accumulation was not observed in any of the sponge incubations (Supplementary Table S3). Nitrate concentrations increased significantly over time in all sponge incubations, suggesting ammonia was oxidized to nitrate without intermediate accumulation of nitrite (Fig. 3A), irrespective of the initial ammonium concentration in the incubation jars (linear regression *P* value = <0.05; Supplementary Table S3). In contrast, no nitrite and nitrate was produced and ammonium concentration remained stable over time in the abiotic control incubations under all three treatment conditions (Fig. 3A; Supplementary Fig. S5; Supplementary Table S3). Sponges kept under “ambient NH_4_^+^” treatment conditions represented an ammonium source (Fig. 3B). In the “5 μM NH_4_^+^” treatment, the NH_4_^+^ production by the sponge holobiont met its consumption rates; however, in the “25 μM NH_4_^+^” treatment, ammonium consumption exceeded the endogenous production rate (Fig. 3B). Moreover, the nitrification rate was significantly higher in the “25 μM NH_4_^+^” treatment (nitrate production rate = 0.1362 μmol g^−1^ ww h ± 0.202 SE) when compared to the “5 μM NH_4_^+^” treatment (nitrate production rate = 0.1083 μmol per g^−1^ ww h ± 0.008 SE; *t*-test Bonferroni-adjusted *P* value = 0.0001), and “ambient NH_4_^+^” (nitrate production rate = 0.096 μmol g^−1^ ww h ± 0.008 SE; *t*-test Bonferroni-adjusted *P* value = 0.0001) condition.

### Phylogeny of dominant nitrifying symbionts

Two high-quality nitrifying symbiont MAGs (>90% completeness and <5% contamination) were recovered, belonging to the archaeal family *Nitrosopumilaceae* and the bacterial order *Nitrospirales*, respectively (Supplementary Table S1)*.*

**Figure 4:**
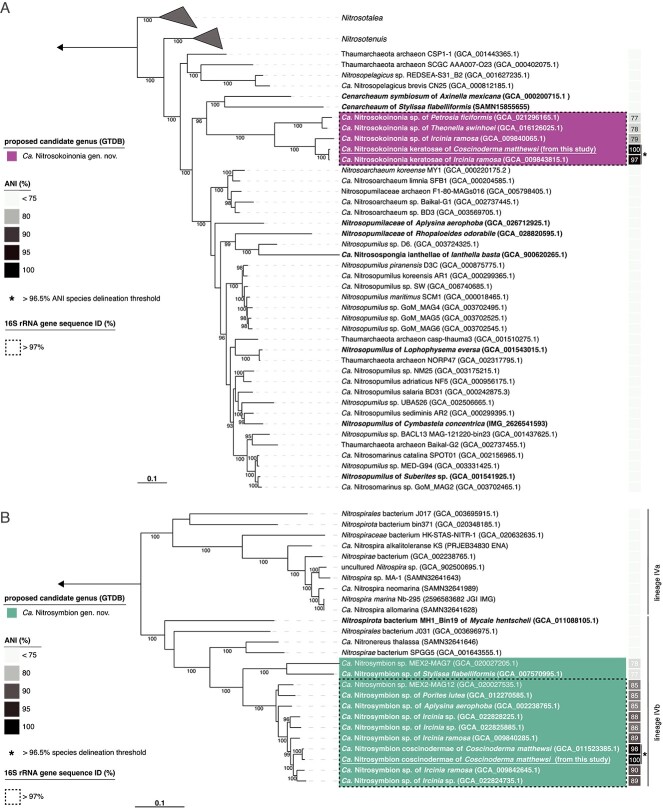
Phylogeny of nitrifying symbionts associated with the sponge *C. matthewsi*. Maximum-likelihood tree of (A) AOA belonging to the family of *Nitrosopumilaceae* and (B) the NOB lineage IV *Nitrospirales*. Both phylogenomic trees are based on concatenated marker proteins identified with checkM v1.1.3 and depict the positions of the recovered MAGs from the sponge *C. matthewsi*. Genomes and MAGs of previously described sponge symbionts are depicted in bold. The proposed candidate genera are highlighted in color. The ANIs of the MAGs from the dominant symbionts associated with the sponge *C. matthewsi* (recovered in this study) to other publicly available genomes and MAGs are displayed as a heatmap. MAGs highlighted with an asterisk (^*^) have an ANI >96.5% (i.e. above the species delineation threshold) with MAGs recovered in this study. The dashed box highlights the boundaries of 16S rRNA gene sequence similarities >97% (see Supplementary Table S1). Numbers at the branches indicate ultrafast bootstrap (*n* = 1000) support. The scale bar corresponds to 0.1 estimated amino acid substitution per site.

In a concatenated marker protein tree, the archaeal MAG was most closely related to four MAGs previously recovered from the marine sponges *Ircinia ramosa, Theonella swinhoei*, and *Petrosia ficiformis* (Fig. 4A)*.* The genome-wide average nucleotide identity (ANI) with these four MAGs ranged from 77% to 97% (Fig. 4A and Supplementary Table S1), and based on the GTDB-tk analysis (Supplementary Table S4), these sponge-associated MAGs (*n* total = 5) form the yet-unnamed Genome Taxonomy Database (GTDB) genus “VYCS01.” Therefore, we propose to name this genus *Candidatus* Nitrosokoinonia gen. nov. (see Taxonomic consideration of *Candidatus* Nitrosokoinonia keratosae gen. nov. sp. nov). Furthermore, based on a species-delineation threshold of 96.5% ANI [72], one MAG (GCA 009843815.1) previously recovered from the closely related sponge *I. ramosa* represents the same species (Fig. 4A). Here, we propose the name *Candidatus* Nitrosokoinonia keratosae gen. nov. sp. nov. (see Taxonomic consideration of *Candidatus* Nitrosokoinonia keratosae gen. nov. sp. nov). Because no 16S rRNA gene was present in either *Ca*. N. keratosae MAG, the 16S rRNA gene amplicons of the two dominant archaeal ASVs (ASV_3dn_o9y and ASV_a8e_mqu) were placed into a full-length 16S rRNA gene tree of publicly available AOA genomes. The best placement location of ASV_3dn_o9y was with other members of the here-described genus *Ca*. Nitrosokoinonia and for the ASV_a8e_mqu with the previously described sponge symbiont *Ca*. Nitrosospongia ianthellae (Supplementary Fig. S6). This suggests that the dominant archaeal ASV_3dn_o9y can be linked to the MAG of *Ca*. Nitrosokoinonia keratosae.

The concatenated marker protein tree of lineage IV *Nitrospirales* showed a clear phylogenetic placement of the MAG described in this study amongst MAGs (n total = 12) of the yet-unnamed GTDB genus “bin75” within the lineage IVb (Fig. 4B). MAGs of this genus are predominantly associated with marine sponges (i.e. *Aplysina aerophoba*, *C. matthewsi*, *I. ramosa*, *Ircinia* sp., and *Stylissa flabelliformis*). One of the MAGs within this genus was previously retrieved from the coral *Porites lutea* [73]. Only 2 of the 12 representative MAGs within this genus were not recovered from an animal host but rather originated from cold seep environments [74], which often harbour encrusting sponges and cold-water corals [75]. However, given that most of the MAGs belonging to this genus were recovered from marine sponges, we propose to name this genus *Candidatus* Nitrosymbion gen. nov. (see Taxonomic consideration of *Ca.* Nitrosymbion coscinodermae gen. nov. sp. nov). Moreover, based on a species-delineation threshold of 96.5% ANI [72], the MAG retrieved in this study represents the same species as one MAG previously assembled from samples of the same sponge species (GCA 011523385.1, Fig. 4B). Thus, we propose the name *Candidatus* Nitrosymbion coscinodermae gen. nov. sp. nov. (see Taxonomic consideration of *Ca.* Nitrosymbion coscinodermae gen. nov. sp. nov). Both *Ca*. N. coscinodermae MAGs contained the 16S rRNA gene, which showed a 100% nucleotide sequence identity with the dominant *Nitrospirales* ASV of the amplicon dataset (ASV_myj_c3n), implying that the *Ca*. N. coscinodermae MAG and the ASV_myj_c3n represent the same symbiont.

### Global occurrence of *Ca*. Nitrosokoinonia and *Ca*. Nitrosymbion

**Figure 5:**
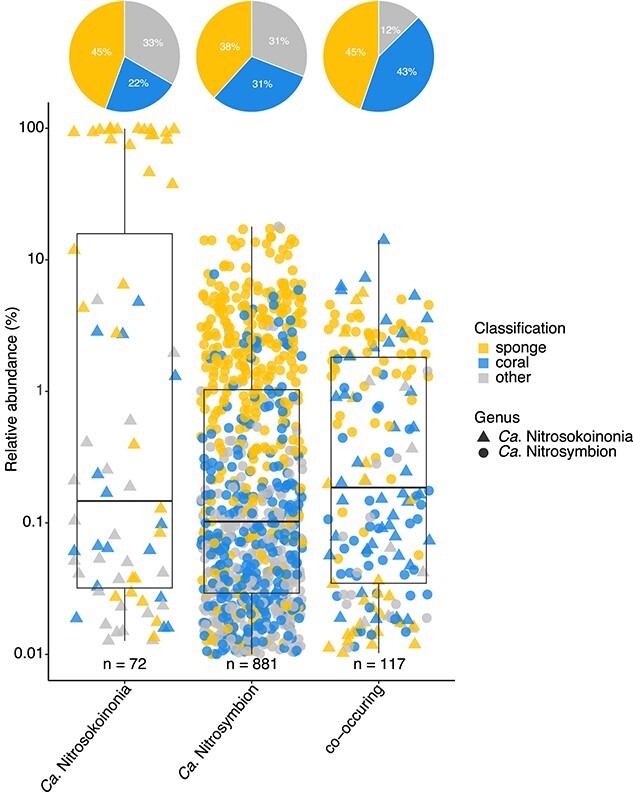
Environmental distribution of *Ca.* Nitrosokoinonia and *Ca.* Nitrosymbion. Relative abundances of the 16S rRNA genes grouped at 97% sequence identity level from publicly available amplicon sequencing datasets obtained from IMNGS. The numbers of the archaeal *Ca*. Nitrosokoinonia might be heavily under- or overestimated in publicly available amplicon datasets depending on the primer selection. Hence, differences in the primer selection might explain the detection discrepancy of the here-described bacterial and archaeal nitrifying genera in publicly available datasets. Each point represents an environmental sample where a closely related organism (>97% sequence similarity) was detected, with a minimal relative abundance of 0.01%. Colors represent the environmental source of the individual samples and shapes represent the respective genus. The *y*-axis is log10-transformed. Pie charts show the relative frequencies (in %) of samples from sponges, corals, and other environments.

The occurrence of the archaeal candidate genus Nitrosokoinonia and the bacterial candidate genus Nitrosymbion in publicly available amplicon datasets was explored using IMNGS [62]. In total, 1070 samples (>97% sequence similarity and >0.01% relative abundance) were identified to contain either a member of *Ca*. Nitrosokoinonia (72 samples), or *Ca*. Nitrosymbion (881 samples), or both (117 samples). Both genera predominantly occur in samples (67% to 88%) of marine sponges and corals (Fig. 5), yet, they were also detected in seawater and sediment samples that were often collected in close proximity to coral reefs (Supplementary Table S2). Overall, this analysis suggests that members of the genera *Ca*. Nitrosokoinonia and *Ca*. Nitrosymbion occur and co-occur in marine sponges and corals around the globe.

**Figure 6:**
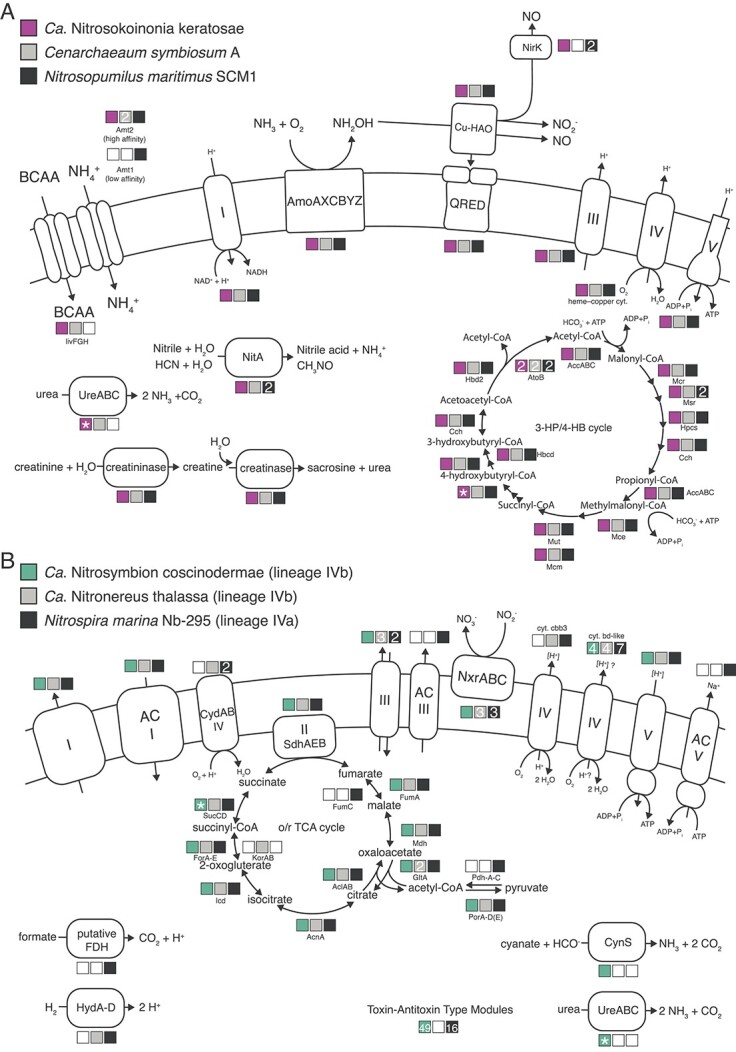
Schematic overview of selected metabolic key pathways. (A) Genomic repertoire of the ammonia-oxidizing archaeal sponge symbionts *Ca*. Nitrosokoinonia keratosae and *Cenarchaeum symbiosum* A as well as the marine *Nitrosopumilus maritimus* SCM1. The illustration depicts key steps of the putative ammonia-oxidation pathway, i.e. the ammonia-monooxygenase enzyme (AmoAXCBYZ), the putative copper-containing hydroxylamine oxidase (Cu-Hao), the putative quinone reductase (QRED), and the nitrite reductase (NirK). It further highlights the electron transport chain (complex I–V) and the 3-hyroxypropionate/4-hydroxybutyrate (3-HP/4-HB) cycle mediating CO_2_ fixation. In addition, selected key functions such as ammonia transporters (Amt1 and Amt2) and BCAA transporters, as well as pathways facilitating the utilization of alternative substrates such as urea (UreABC), nitrile/cyanide (NitA), and creatinine (creatininase and creatinase). B) Genomic repertoire of the NOB sponge symbiont *Ca*. Nitrosymbion coscinodermae, as well as the marine *Ca*. Nitronereus thalassa and *Nitrospira marina* Nb-295. The illustration depicts hallmark genes of NOB such as the nitrite oxidoreductase (NxrABC), the membrane-bound respiratory chain (complex I–V) including the alternative complexes (AC I–V), and the oxidative and reductive tricarboxylic acid (o/r TCA) cycles. In addition, selected key functions such as TA modules, formate metabolization (putative FDH), putative hydrogen oxidation (HydA-D; group 3b [NiFe] hydrogenase), and cyanate (CynS) and urea (UreABC) utilization. Colored boxes indicate the presence of genes coding for the shown proteins in the respective genomes. If more than one copy of a gene was present, the copy number of this gene is displayed in the box. Asterisk (^*^) indicates that genes were missing in the MAG of the species representative but were present in the other MAG(s) of the newly described genus.

### Genome characteristics of the novel candidate genera

MAGs of the archaeal genus *Ca*. Nitrosokoinonia are on average 1.61 Mbp (±0.18 Mbp SD) in size and have a median GC content of 51.90% (±8.78% SD; Supplementary Table S1), which is smaller than the genome size of other sponge-associated AOA genomes (1.87 Mbp ± 0.30 Mbp SD). Generally, most described sponge-associated AOA have larger genomes (1.77 Mbp ± 0.28 Mbp SD) and a higher GC content (50.00% ± 12.79% SD) than free-living relatives (1.47 Mbp ± 0.25 Mbp SD and 35.40% ± 3.96% SD) of the family *Nitrosopumilaceae* (Supplementary Table S1). A pan-genome analysis including the new genus *Ca*. Nitrosokoinonia (*n* = 5), other sponge-associated AOA (*n* = 8), and closely related free-living AOA (*n* = 29; Supplementary Table S1) showed that a considerable number of unique orthologs are exclusively found in *Ca*. Nitrosokoinonia (34) and other sponge-associated AOA (135) genomes, or were shared amongst all symbiont genomes (29; Supplementary Fig. S7A and Supplementary Table S5). For example, the unique orthologs include genes encoding for lipid A (endotoxin) biosynthesis, eukaryotic-like-proteins (Toll-interleukin-1 receptor [TIR]–like domain DUF1863), and restriction–modification systems (including a type II restriction endonuclease).

Genomes of the genus *Ca*. Nitrosymbion are on average 2.36 Mbp (±0.29 Mbp SD) in size and have a GC content of 56.90% (±1.63% SD; Supplementary Table S1). In general, the average genome size of all lineage IVb *Nitrospirales* is smaller (2.44 Mbp ± 0.57 Mbp SD) when compared to genomes of the lineage IVa (3.84 Mbp ± 0.12 Mbp SD; Supplementary Table S1). However, within Lineage IVb, there is a large range in genome sizes. Some of the *Ca*. Nitrosymbion MAGs have the smallest genomes within this lineage, whereas the only cultivated representative, *Ca*. *Nitronereus thalassa*, has an unusually large genome (4.02 Mbp; Supplementary Table S1). A pan-genome analysis including the new genus *Ca*. Nitrosymbion (*n* = 12), other members of lineage IVb (including *Ca*. N. thalassa; *n* = 4), and members of lineage IVa (*n* = 9; Supplementary Table S1) showed that 385 identified orthologs are exclusively present in MAGs of the genus *Ca*. Nitrosymbion (Supplementary Fig. S7B and Supplementary Table S6). For example, the unique orthologs include genes that encode for multiple toxin–antitoxin (TA) modules, bacteriocin protection mechanism, and a solute/sodium symporter.

### Genomic potential of *Ca.* Nitrosokoinonia keratosae gen. nov. sp. nov.

The representative MAG of *Ca*. Nitrosokoinonia keratosae (97.57% completeness and 0.00% contamination) contains all the hallmark genes for chemolithoautotrophic ammonia oxidation and CO_2_ fixation (Fig. 6A and Supplementary Table S7). This includes genes encoding for all known and putative subunits of the ammonia monooxygenase gene cluster (*amo*AXBCYZ), the 3-hyroxypropionate/4-hydroxybutyrate (3HP/4HB) cycle for CO_2_ fixation, and the electron transport chain (complex I–IV). Like all other AOA genomes of the family *Nitrosopumilaceae*, except for *Cenarchaeum symbiosum*, *Ca*. N. keratosae also encodes for the NO-forming Cu-containing nitrite reductase (*nirK*), which has been hypothesized to play a critical role in the currently postulated archaeal ammonia oxidation pathways [76, 77]. Furthermore, *Ca*. N. keratosae encodes for the candidate enzyme “*Cu-Hao*,*”* which has been suggested to be putatively involved in hydroxylamine oxidation [78, 79]. The *Ca*. N. keratosae genome also encodes for a high-affinity ammonia transporter (*amt*2) [80] and, like almost all other sponge-associated AOA genomes, lacks the low-affinity ammonia transporter (*amt1*) and only contains one nitrogen regulatory protein PII gene. In the genomes of their free-living relatives, *amt1* transporters and additional copies of the nitrogen regulatory protein PII genes can be found. *Ca*. N. keratosae might be able to directly use sponge-derived urea, nitriles/cyanides, and creatinine but not cyanate as alternative ammonia sources. Its genome contains a urease gene cluster (*ureABC*), a gene encoding for nitrilase/cyanide hydratase (*nitA*), and for a creatinase/creatinine amidohydrolase as well as for creatinase/creatin amidohydrolase (Fig. 6A). Additionally, the genome of *Ca*. N. keratosae encodes for branched-chain amino acid (BCAA) transporters (*livFGM*)—a genomic feature unique to sponge-associated AOA [17].

### Genomic potential of *Ca.* Nitrosymbion coscinodermae gen. nov. sp. nov

The genome of the nitrite-oxidizing sponge symbiont *Ca*. Nitrosymbion coscinodermae (completeness 98.64% and contamination 3.86%) encodes for all hallmark genes for chemolithoautotrophic nitrite oxidation and CO_2_ fixation. This includes genes for the known subunits of nitrite oxidoreductase (*nxrABC*), the oxidative (oTCA) and reductive (rTCA) tricarboxylic acid cycles, and the membrane-bound respiratory chain (Fig. 6B and Supplementary Table S8). Similar to other genomes of the order *Nitrospirales*, *Ca*. N. coscinodermae encodes for four putative cytochrome *bd*-like oxidases (complex IV); however, it lacks the high-affinity, heme-copper cytochrome *cbb_3_*-type terminal oxidases found in some other lineage IV *Nitrospirales* (Fig. 6B and Supplementary Table S8). Moreover, the genome of *Ca*. N. coscinodermae also lacks the putative alternative complexes (AC) III and V (Fig. 6B and Supplementary Table S8).

The order *Nitrospirales* likely emerged from anaerobic ancestors [69]. Based on the observed genome adaptations, we propose that *Ca*. N. coscinodermae, like all other members of the order *Nitrospirales*, has adapted to life under oxygenated conditions. For example, the *Ca*. N. coscinodermae genome encodes for the presumably oxygen-tolerant form of pyruvate:ferredoxin oxidoreductase (*porA-D*) [81] and the five subunits of oxoglutarate:ferredoxin oxidoreductase (*forA-E*), key enzymes of the o/rTCA cycle that are universally present in genomes of the order *Nitrospirales*. In contrast to the closely related *Ca*. Nitronereus thalassa [82], the *Ca*. N. coscinodermae genome lacks an isoenzyme Kor (*korAB*), which was previously described to facilitate growth under anaerobic conditions [83].

Alternative electron donors, such as hydrogen and formate, have been described to be used by some *Nitrospirales* [81, 84–86]. In the genome of *Ca*. N. coscinodermae, putative formate dehydrogenase genes (putative FDH) or genes related to hydrogen metabolism (3b [NiFe] hydrogenase, 2a [NiFe] hydrogenase, and related genes involved in hydrogenase maturation) were not detected (Fig. 6B and Supplementary Table S8). Thus, alternative metabolisms based on formate and hydrogen seem unlikely. Moreover, the *Ca*. N. coscinodermae genome also lacks the canonical cytochrome *bd* quinol oxidase (*cydAB*, complex IV), which accepts electrons from electron donors with lower reduction potential than nitrite and may play a role in the oxidative stress response in other *Nitrospirales* [81, 84–86].

Based on its genomic repertoire, *Ca*. N. coscinodermae may be able to take up small dissolved organic nitrogen compounds, such as cyanate and urea, for nitrogen assimilation. The genome of *Ca*. N. coscinodermae encodes for the enzymes cyanase (*cynS*) and urease (*ureABC*), both of which can cleave ammonia from the original substrate and release CO_2_. Thus, *Ca*. N. coscinodermae potentially indirectly gains energy from cyanate and urea via reciprocal feeding [85, 87] with the AOA symbiont population within the sponge holobiont. The genome of *Ca*. N. coscinodermae as well as other members of the genus *Ca*. Nitrosymbion is enriched in genes encoding for TA modules, especially of type II and type IV, which may reflect an adaptation to a life in close proximity to other microbes, a defence mechanism against phages and/or playing a role in evading host phagocytosis [88].

## Discussion

Marine sponge microbiomes can be highly diverse [12, 89]. Yet, nitrification appears to be a ubiquitous functional trait in these symbiotic communities (e.g. [6, 21, 22, 90]). In this study, we characterize two novel genera of nitrifiers, *Ca*. Nitrosokoinonia and *Ca*. Nitrosymbion, which frequently co-occur in the microbiome of many marine sponges and corals (Fig. 5 and Supplementary Table S2). The prevalent symbiotic lifestyle and co-occurrence of *Ca*. Nitrosokoinonia and *Ca*. Nitrosymbion may indicate a co-evolutionary relationship between the two symbiont lineages and their shared habitat preferences; further research is needed to better understand the effect of both symbiont lineages on each other’s evolutionary trajectory.

Vertical symbiont transmission is common in marine sponges [11, 13] and has also been observed for nitrifying symbionts [91]. Viviparous sponge species (“brooders”) predominantly transfer their symbionts vertically from one generation to the next [11]. *C. matthewsi* is a viviparous sponge [92], and we detected symbionts in high densities in the mesohyl of freshly released larvae (Fig. 2). The nitrifying symbiont community associated with freshly released larvae was dominated by *Ca*. N. keratosae and *Ca*. N. coscinodermae (Fig. 1). MAGs with the highest ANI (>88% ANI) to the representative symbionts of *C. matthewsi* were associated with the closely related viviparous sponge *I. ramosa* (Fig. 4 and Supplementary Table S1). Symbionts belonging to the novel genera are not restricted to viviparous sponge species of the order Dictyoceratida but can also be detected in tissue samples of oviparous sponges such as *Aplysina aerophoba* (Fig. 4 and Supplementary Table S3). Larvae of oviparous sponges have been described to already contain symbionts during their development outside of the mother sponge [91, 93]. Because all currently known sponge larvae are lecithotrophic, i.e. they do not feed during their short planktonic life stage [94], the symbionts of the herein described genera are likely vertically transmitted in both viviparous and oviparous sponges. However, the precise transmission strategy of both symbiont lineages remains to be determined.

The ammonia-oxidizing archaeal symbiont *Ca*. N. keratosae dominated the larval microbiome (Fig. 1). The overrepresentation of AOA in the larval microbiome may suggest that they play an essential role in the early life stages of a sponge. For example, vertically inherited AOA of the genus *Ca*. Nitrosokoinonia may induce early metamorphosis of their sponge host via the production of nitric oxide (NO). Nitric oxide is a well-known signalling molecule in marine invertebrates [95]. For instance, in the sponge *Amphimedon queenslandica,* nitric oxide has been shown to trigger larval settlement and metamorphosis [96]. Nitric oxide production by AOA (Fig. 6A) in sponge larvae could potentially induce settlement and therefore either help to sense a suitable substrate (i.e. ammonia and oxygen availability) or function as an indicator of nitrogenous waste accumulation. Elucidating the function and nitrification activity of AOA symbionts in sponge larvae and their ability to interfere with the eukaryotic signalling pathway of their host is an interesting avenue for further research.

Nitrification activity was measured in the adult sponge holobiont. Linking physiological data (Fig. 3) with the genomic potential of *Ca*. N. keratosae and *Ca*. N. coscinodermae (Fig. 6) indicates that the dominant nitrifying symbionts actively contribute to the removal of endogenously produced ammonia from the sponge holobiont. However, based on 16S rRNA gene amplicon sequencing (Fig. 1), *Ca*. N. keratosae and *Ca*. N. coscinodermae are not the only nitrifying symbionts residing within the sponge holobiont. Addressing microdiversity and niche differentiation in terms of spatial distribution and substrate utilization will be crucial to understanding co-occurrence of functionally redundant symbionts within a single sponge host. It also remains to be determined how symbiont shuffling and/or switching of functionally redundant symbionts can affect the phenotypic plasticity of the holobiont and, thus, facilitate adaptation.

Nitrogenous waste products of the sponge holobiont fuel the symbiont-mediated nitrification activity. Nitrification rates of the *C. matthewsi* microbiome are within the range of previously measured nitrification rates of other high-microbial abundance sponges (e.g. [21, 22, 26, 97]), seem to rely on endogenous ammonia sources, and are not drastically affected by external ammonia concentrations (Fig. 3). Similar observations have recently been described for the Mediterranean sponge *Chondrosia reniformis*, where exogenous ammonia concentrations did not significantly affect the ammonia and oxygen consumption nor the exhaled nitrate concentrations [98]. Ammonia assimilation in AOA is regulated by nitrogen regulatory proteins PII, which are overrepresented in free-living AOA genomes [99]. Nitrogen regulatory proteins receive information about the carbon/nitrogen ratio and energy status of the cell and regulate ammonia uptake and utilization in response to changes in extracellular nitrogen availability [100]. Because sponge-associated AOA are universally equipped with the high-affinity ammonia transporter (*amt2*) and reduced copy numbers of nitrogen regulatory protein PII genes (Supplementary Table S7), the symbiotic AOA seem to be adapted to low but stable ammonium concentrations. Besides ammonium, alternative nitrogenous substrates such as urea, cyanide, creatinine, and BCAAs may play a crucial role in fuelling the ammonia oxidation rate of *Ca*. Nitrosokoinonia, as they are ubiquitously equipped with genes encoding for urease (*ureABC*), nitrilase/cyanide hydratase (*nitA*), creatinase (*arfB*), and BCAA transporters (Fig. 6A). Nitrogenous terpenoids, which feature nitrogen-containing functional groups like isonitriles, isothiocyanates, and formamides that originate from inorganic cyanide, are a secondary metabolite class ubiquitously produced by marine sponges [101]. Thus, sponge-produced nitrogenous terpene [101], and other nitrogen-containing secondary metabolites [102], as well as creatin/creatinine [36] might function as additional ammonia sources. Moreover, *Ca*. Nitrosymbion genomes also encode genes for enzymes involved in utilizing simple organic nitrogen compounds—urease (*ureABC*) and cyanase (*cynS;*  Fig. 6B). This suggests nitrite-oxidizing symbionts of the here-described genus *Ca*. Nitrosymbion can convert urea and cyanate to ammonia and CO_2_, providing an additional energy source to the resident AOA symbiont population within the holobiont and receiving nitrite in return. Reciprocal feeding would be particularly relevant if the co-occurring AOA population lacks a urease [85, 87], which is currently uncertain (Fig. 6B).

Overall, this study characterizes two new genera of AOA and NOB that frequently occur and co-occur in marine sponges and corals. The predominant association of these genera with basal metazoan hosts and the vertical transmission mode suggest that both genera are well adapted to their symbiotic lifestyle and may even have co-evolved alongside their hosts. Furthermore, the high number of unique orthologs suggest an immense source of genetic novelty. We therefore propose that both genera, *Ca*. Nitrosokoinonia and *Ca*. Nitrosymbion, are an ideal model system to study co-evolution and co-diversification of symbionts associated with marine sponges and corals. Marine sponge holobionts are currently emerging as an interesting model system due to their complex microbe–microbe and host–microbe interactions [20]. Here, we particularly emphasize the importance of studying niche partitioning within the sponge holobiont (e.g. nitrification/denitrification activity along oxygen microgradients), single-cell interactions amongst holobiont members, and the effect of symbiont-produced metabolites on the host-signalling pathways.

### Taxonomic consideration of *Ca.* Nitrosokoinonia keratosae gen. nov. sp. nov

ni'tro.so.koi.no'ni.a L. n. nitroso: nitrosus, full of natron, here intended to mean nitrous; Gr. fem. n. koinonia: refers to concepts such as fellowship, joint participation, partnership; L. fem. n. Nitrosokoinonia partner that consumes ammonia. N.L. n. keratosa: subclass of Demospongiae comprising horny sponges with a spongin skeleton and without spicules.

An ammonia-oxidizing, nitrite-producing archaeon associated with keratose sponges. Phylogenetically affiliated with the order *Nitrosopumilales*, phylum *Thermoproteota* (syn. *Crenarchaeota*). The metagenome-assembled genome (MAG) is 97.57% complete and has 0.00% contamination. It consists of 162 scaffolds, with a total of 1 505 883 bp. The DNA G + C content is 45.56%. *Ca*. Nitrosokoinonia keratosae was recovered from the tissue of two closely related marine sponge species (*I. ramosa* and *C. matthewsi)*, collected at Great Barrier Reef in Queensland (Australia).

### Taxonomic consideration of *Ca.* Nitrosymbion coscinodermae gen. nov. sp. nov

Ni.tro.sym·bi·on L. n. nitrum: nitrate, Gr. n. symbion: living together; L. fem. n *Nitrosymbion* nitrate-forming symbiont. cos.cino.dér.mae Gr. fem. n. koskinon: sieve, Gr. fem. n dermae: skin; Gr. fem. n. a sieve-like skin, in this case referring to the sponge genus *Coscinoderma*; a nitrite-oxidizing, nitrate-forming symbiont obtained from a marine sponge. Phylogenetically affiliated with the order *Nitrospirales*, phylum *Nitrospirota*. The MAG is 98.64% complete and has 3.86% contamination. It consists of 38 scaffolds, with a total of 2 506 777 bp. The DNA G + C content is 56.70%. *Ca*. Nitrosymbion coscinodermae was recovered from the tissue of the marine sponge *C. matthewsi*, collected at the in-shore coral reef location Falcon Island (Great Barrier Reef, Queensland, Australia).

## Supplementary Material

Supplementary_Materials_wrae069

Supplementary_TableS1-S8_wrae069

## Data Availability

The assembled genomes, raw sequencing reads, and amplicon sequencing (16S rRNA gene, 28S rRNA gene and CO1 gene) data were generated under the JMF project ID JMF-2203-05 and JMF-2305-01, and are available under the NCBI BioProject PRJNA1037309. Furthermore, all assembly IDs of publicly available genomes and MAGs used within this study are summarized in Supplementary Table S1. Nitrification data, including NH_4_^+^, NO_2_^−^, and NO_3_^−^ concentrations, are available in Supplementary Table S3.
